# Association of early postnatal transfer and birth outside a tertiary hospital with mortality and severe brain injury in extremely preterm infants: observational cohort study with propensity score matching

**DOI:** 10.1136/bmj.l5678

**Published:** 2019-10-16

**Authors:** Kjell Helenius, Nicholas Longford, Liisa Lehtonen, Neena Modi, Chris Gale

**Affiliations:** 1 Department of Paediatrics and Adolescent Medicine, Turku University Hospital, Turku, Finland; 2 Department of Clinical Medicine, University of Turku, Turku, Finland; 3 Section of Neonatal Medicine, Department of Medicine, Chelsea and Westminster campus, Imperial College London, London SW10 9NH, UK

## Abstract

**Objective:**

To determine if postnatal transfer or birth in a non-tertiary hospital is associated with adverse outcomes.

**Design:**

Observational cohort study with propensity score matching.

**Setting:**

National health service neonatal care in England; population data held in the National Neonatal Research Database.

**Participants:**

Extremely preterm infants born at less than 28 gestational weeks between 2008 and 2015 (n=17 577) grouped based on birth hospital and transfer within 48 hours of birth: upward transfer (non-tertiary to tertiary hospital, n=2158), non-tertiary care (born in non-tertiary hospital; not transferred, n=2668), and controls (born in tertiary hospital; not transferred, n=10 866). Infants were matched on propensity scores and predefined background variables to form subgroups with near identical distributions of confounders. Infants transferred between tertiary hospitals (horizontal transfer) were separately matched to controls in a 1:5 ratio.

**Main outcome measures:**

Death, severe brain injury, and survival without severe brain injury.

**Results:**

2181 infants, 727 from each group (upward transfer, non-tertiary care, and control) were well matched. Compared with controls, infants in the upward transfer group had no significant difference in the odds of death before discharge (odds ratio 1.22, 95% confidence interval 0.92 to 1.61) but significantly higher odds of severe brain injury (2.32, 1.78 to 3.06; number needed to treat (NNT) 8) and significantly lower odds of survival without severe brain injury (0.60, 0.47 to 0.76; NNT 9). Compared with controls, infants in the non-tertiary care group had significantly higher odds of death (1.34, 1.02 to 1.77; NNT 20) but no significant difference in the odds of severe brain injury (0.95, 0.70 to 1.30) or survival without severe brain injury (0.82, 0.64 to 1.05). Compared with infants in the upward transfer group, infants in the non-tertiary care group had no significant difference in death before discharge (1.10, 0.84 to 1.44) but significantly lower odds of severe brain injury (0.41, 0.31 to 0.53; NNT 8) and significantly higher odds of survival without severe brain injury (1.37, 1.09 to 1.73; NNT 14). No significant differences were found in outcomes between the horizontal transfer group (n=305) and controls (n=1525).

**Conclusions:**

In extremely preterm infants, birth in a non-tertiary hospital and transfer within 48 hours are associated with poor outcomes when compared with birth in a tertiary setting. We recommend perinatal services promote pathways that facilitate delivery of extremely preterm infants in tertiary hospitals in preference to postnatal transfer.

## Introduction

About one in 20 preterm infants in high income countries are born at less than 28 weeks’ gestation (5.7% in the United Kingdom and 7% in the United States)^1^ ^2^; these extremely preterm infants are at high risk of death and neonatal morbidity, such as periventricular and intraventricular haemorrhage^3^ ^4^ and long term disability.^5^ ^6^ Caring for extremely preterm infants is complex, and previous studies have shown optimal outcomes when care is provided in tertiary hospitals.^7^ ^8^ ^9^ ^10^ ^11^ Such hospitals have a delivery unit equipped and staffed to provide a full range of perinatal and obstetric care for mothers, and a neonatal intensive care unit in which extremely preterm infants can be stabilised and receive ongoing care. Several national guidelines recommend a regionalised model of care for extremely preterm infants, where the goal is to deliver these infants in tertiary hospitals.^12^ ^13^ ^14^

When women at risk of extremely preterm delivery present at non-tertiary hospitals, transfer to a tertiary hospital can occur either before delivery (prenatal or in utero transfer) or after delivery following stabilisation of the infant in the non-tertiary hospital (postnatal transfer). In England, postnatal transfers increased after the reorganisation of care into regional networks in 2007.^15^ ^16^ During 2009-10 less than 50% of infants born at 27 and 28 weeks’ gestation in England were delivered in high volume (>2000 annual neonatal intensive care days) hospitals, whereas reports from New South Wales, Australia, and Finland showed that 85% and 95%, respectively, of infants born at less than 28 weeks’ gestation were delivered in tertiary hospitals.^17^ ^18^ ^19^ Although historical studies show that preterm infants who underwent postnatal transfer had higher rates of adverse outcomes than infants born in tertiary hospitals,^20^ ^21^ ^22^ recent studies have shown equivocal results on the association between early postnatal transfer and outcomes.^23^ ^24^ ^25^ ^26^ ^27^ It is unclear whether the association between postnatal transfer and adverse outcomes persists in the context of modern neonatal care and dedicated neonatal transfer services. Harms associated with postnatal transfer might relate to suboptimal stabilisation at a non-tertiary hospital, the transfer of sicker infants, or the transfer itself. This is relevant to the organisation of perinatal health services because early postnatal transfers are increasingly common in the UK and other high income countries following the introduction of highly specialised neonatal transfer services.^15^ ^28^

We examined the association between outcomes and early postnatal transfer and ongoing non-tertiary neonatal care in extremely preterm infants born in England. We also sought to separate the possible effects of postnatal transfer from those of delivery and initial stabilisation in a non-tertiary hospital. Because these research questions are not amenable to a randomised controlled trial, we conducted an observational study applying propensity score matching to form groups for comparison with near identical distributions of background and potential confounder variables. Our hypothesis was that mortality and severe brain injury would be higher in transferred infants compared with non-transferred infants born in tertiary hospitals.

## Methods

We performed a retrospective cohort study of all infants born before 28 weeks’ gestation and admitted to neonatal units in England from 1 January 2008 to 31 December 2015. Data were extracted from the UK National Neonatal Research Database (NNRD), which holds deidentified descriptive (eg, birth weight), daily (eg, daily respiratory support), episodic (eg, surgery), and diagnostic data extracted from routine electronic health records for all infants admitted to national health service neonatal units in England from 2012 to present, and most infants from 2008 to 2011. In England, neonatal care for extremely preterm infants is not provided outside the NHS. Data are cleaned before inclusion in the NNRD; records with implausible data configurations are queried and, if necessary, corrected by the treating clinicians. A formal comparison of NNRD data with case record forms from a multicentre, randomised controlled trial^29^ showed high data agreement and a high coverage of infants included in the NNRD compared with national statistics; for infants born at 25 gestational weeks or more the NNRD covers close to 100% of national live births and about 70% and 90% of infants born at 23 and 24 gestational weeks, respectively. The NNRD contains a clinical dataset (the National Neonatal Data Set), which is an approved NHS information standard for England and contained within the NHS Data Dictionary (see data items at www.datadictionary.nhs.uk/data_dictionary/messages/clinical_data_sets/data_sets/national_neonatal_data_set/national_neonatal_data_set_-_episodic_and_daily_care_fr.asp?shownav=1.)^30^

English neonatal units are organised in regional networks of non-tertiary and tertiary neonatal units. The intention is for infants requiring high level care to receive it in a tertiary neonatal unit, with step-down transfer to a non-tertiary neonatal unit within the same network when appropriate. Neonatal units in English hospitals are classified as special care baby units, local neonatal units, and neonatal intensive care units^31^; in this study neonatal intensive care units are referred to as tertiary neonatal units. These definitions differ slightly from those of the American Academy of Paediatrics. Local neonatal units in the UK are generally expected to be able to care for uncomplicated, singleton infants from 27 weeks’ gestation onwards and twins or higher order multiples from 28 weeks’ gestation onwards and to arrange in utero transfer when delivery is expected at lower gestational ages or postnatal transfer after delivery of an infant at a lower gestational age. Variation exists within England, however, and many networks apply a gestational age of 28 weeks as the threshold for referral from a hospital with a local neonatal unit to one with a tertiary neonatal unit. Special care baby units are expected to be able to care for uncomplicated, singleton infants of 32 weeks’ gestation onwards and to transfer infants at lower gestational ages. One aim of this organisational framework is to deliver extremely preterm infants at hospitals with tertiary neonatal units. Since the introduction of a networked model of neonatal care, however, postnatal transfers in England have become more frequent.^15^ Dispatching resuscitation teams to assist at the delivery of extremely preterm deliveries is not standard practice in England.

We defined four groups of infants based on birth hospital and transfer status at 48 hours, selected a priori based on previous work.^15^ ^25^ The upward transfer group comprised infants born in a hospital with a local neonatal unit and transferred to a tertiary hospital within 48 hours. The non-tertiary care group comprised infants born in a hospital with a local neonatal unit and not transferred within 48 hours; this group was not prespecified in the protocol and analysis was undertaken when it became apparent that many infants were defined as belonging within this group. The horizontal transfer group comprised infants born in a tertiary hospital and transferred within 48 hours to a different tertiary hospital for non-clinical reasons such as insufficient capacity. The control group comprised infants born in a tertiary hospital and not transferred within 48 hours.

For the primary analysis we identified matched groups of infants from non-tertiary care, upward transfer, and control. Comparisons undertaken between these three matched groups took place between infants in the upward transfer and control groups to evaluate the associations between birth at and initial stabilisation in a hospital with a local neonatal unit and early postnatal transfer; between infants in the non-tertiary care and control groups to evaluate the associations between birth, initial stabilisation, and care in a hospital with a local neonatal unit without early postnatal transfer; and between infants in the upward transfer and non-tertiary care groups to evaluate the association between early postnatal transfer compared with continuing care in a local neonatal unit, among infants born in a local neonatal unit.

In a secondary analysis we identified matched groups of infants in horizontal transfer and control groups to evaluate the association between early postnatal transfer and initial stabilisation in a tertiary hospital (to separate postnatal transfer from initial stabilisation at a lower intensity hospital).

The study protocol was sent to all English neonatal units on 31 August 2016 and published on the National Neonatal Research Database website (www.imperial.ac.uk/neonatal-data-analysis-unit/our-research/past-research-projects/) before data extraction (see supplementary file). We deviated from the study protocol by limiting analysis to infants born in England because of a high rate of missing data in infants born in Scotland and Wales and transferred to or from neonatal units that did not contribute data to the NNRD in the study period.

We extracted data from the NNRD on the variables gestational age, sex, multiplicity, maternal smoking, mode of delivery, use of antenatal steroids, year and month of first neonatal admission, network of first neonatal admission, birth weight z score,^32^ Apgar scores at one and five minutes after birth, and surfactant administered in the delivery room. These variables were included in the propensity analysis on which matching was based. Infants who died in the delivery room or were stillborn were excluded as these are incompletely captured in the NNRD. Infants with a diagnosis of trisomy 13, 18, or 21 or severe congenital malformations requiring early surgical intervention were excluded (see supplementary file) because they are more likely to be transferred or to receive palliative care, and these conditions are associated with adverse outcomes. Infants for whom the indication for transfer was cardiac care or surgery were excluded for the same reason. We also excluded infants with missing data on gestational age, sex, or birth weight because these variables were essential for the propensity matching and were likely to have a large impact on the outcomes. Data on infants with a birth weight z score greater than 4 or less than −4 were excluded as improbable. The outcomes were death before discharge from neonatal care, severe brain injury, and their combination: survival without severe brain injury. Severe brain injury was defined as grade 3 or 4 periventricular or intraventricular haemorrhage,^33^ porencephalic cysts, post-haemorrhagic hydrocephalus, or cystic periventricular leucomalacia on ultrasound scan; a preterm component of the UK Department of Health definition of brain injuries occurring during or soon after birth.^34^ ^35^ Infants with no recorded ultrasound scan and no diagnosis of brain injury were coded as unknown for severe brain injury and dropped from the analyses of severe brain injury. Infants with missing data on mortality were dropped from the analysis on death before discharge. Infants missing relevant data for the analysis of survival without severe brain injury were dropped from the analysis of survival without severe brain injury—for example, infants who survived but had missing data on severe brain injury.

Detailed definitions of covariates and outcomes are provided in the supplementary file. The number needed to treat (NNT) was defined as the number of infants needed to be delivered in a tertiary hospital to avoid one case of the outcome (death, severe brain injury, or death or severe brain injury).

### Statistical analysis

Data were analysed with statistical software packages R (version 3.2.5) and IBM SPSS (version 21.0. IBM; Armonk, NY) using the potential outcomes framework. This involved forming matched within treatment groups that were well balanced on the background variables. Matching was accomplished by propensity analysis, which entailed fitting a logistic regression of the treatment assignment (a variable that indicates upward transfer, non-tertiary care, or control) as the outcome and all the available background variables as the covariates (table 1, see full list in supplementary table 1). The regression model was supplemented by adding interactions of covariates one at a time and selecting the model with superior balance. The output of this analysis is a set of propensity scores. To reduce residual confounding we then trimmed the propensities by excluding infants with extreme propensities. The three largest groups of infants (control, upward transfer, and non-tertiary care) were matched 1:1:1, replicated 50 times, forming triplets of infants with one infant from each group.^36^ Caliper matching based on the logits of propensities was applied with the caliper width set to 0.1. Each triplet consisted of one infant from the upward transfer group, one from the non-tertiary care group, and one from the control group, born in the same year and regional network. The triplets were also matched on sex, gestational week, and use of antenatal steroids, which were regarded as principal covariates. The success of the matching process is illustrated by standardised differences of the background variables across each group before and after matching. No universally accepted limits exist for how small the standardised differences should be to indicate a good match, but one study suggested no concern for standardised differences between −0.2 and 0.2, and for large study samples for standardised differences between −0.1 and 0.1.^36^ The supplementary file provides a detailed description of the matching process. The methods are described in the literature.^37^ ^38^ ^39^ ^40^ Birth weight z scores are not defined for infants born less than 23 gestational weeks because there are no relevant UK reference values. These infants were matched separately, using birth hospital level, sex, use of antenatal steroids, and birth weight (within 30 g). After we had carried out matching in the three intervention groups, we estimated the differences in outcomes using the two tailed *t* test.

**Table 1:** Background characteristics before propensity score matching of extremely preterm infants (<28 gestational weeks) born in England in 2008 to 2015, by hospital of birth and transfer status at 48 hours of age. Values are numbers (percentages) unless stated otherwise

Characteristics	Controls (n=10 866)	Upward transfer (n=2158)	Standardised difference*	Non-tertiary care (n=2668)	Standardised difference†	Standardised difference‡
Median (interquartile range) gestational weeks	26.0 (24.9-27.0)	25.6 (24.6-26.4)	−0.21	27.0 (26.3-27.6)	0.51	−0.70
Mean (SD) birth weight (g)	807 (188)	797 (172)	0.06	931 (193)	0.50	−0.50
Mean (SD) birth weight z score	−0.20 (0.89)	−0.03 (0.82)	0.22	0.02 (0.89)	0.25	−0.05
Boys	5799 (53.4)	1207 (55.9)	−0.03	1463 (54.8)	−0.03	−0.02
Multiple birth	2995 (27.6)	497 (23.1)	−0.09	556 (20.8)	−0.15	0.03
Missing	2 (0)	2 (0)		0 (0)		
Smoking in pregnancy	1733 (19.5)	418 (22.1)	0.08	503 (21.7)	0.04	−0.02
Missing	1998 (18.4)	263 (12.2)		349 (13.1)		
Caesarean delivery	4028 (40.1)	680 (32.9)	0.16	1208 (48.5)	0.21	0.23
Missing	819 (7.5)	93 (4.3)		177 (6.6)		
Surfactant during resuscitation	9780 (94.0)	2035 (97.3)	0.08	2446 (93.7)	0.01	0.11
Missing	466 (4.3)	66 (3.1)		58 (2.2)		
Antenatal steroids	9897 (92.4)	1714 (80.3)	−0.25	2255 (86.5)	−0.12	−0.11
Missing	153 (1.4)	24 (1.1)		60 (2.2)		
Apgar score <3 at 1 min	1847 (19.5)	467 (23.7)	−0.10	409 (17.1)	−0.06	0.12
Missing	1392 (12.8)	186 (8.6)		275 (10.3)		
Apgar score <3 at 5 min	385 (4.1)	101 (5.2)	−0.02	80 (3.4)	−0.02	0.05
Missing	1426 (13.1)	215 (10.0)		331 (12.4)		

Upward transfer=infants born in hospitals with local neonatal units and transferred to tertiary hospitals within 48 hours of birth; non-tertiary care=infants born in hospitals with local neonatal units and not transferred within 48 hours of birth; controls=infants born in tertiary hospitals and not transferred within 48 hours of birth.

* Controls versus upward transfer group.

† Controls versus non-tertiary care group.

‡ Upward transfer group versus non-tertiary care group.

The horizontal transfer group was much smaller than the other three groups and therefore could not be incorporated into the three way matching; instead, we matched these infants separately to controls using the same principles, but by matching one infant in the horizontal transfer group to five infants from the control group. The outcomes were assessed in a similar way, using the two tailed *t* test for estimating the differences in outcomes between the two groups.

### Sensitivity analyses

We performed two further paired, matched analyses between infants in the upward transfer group and control group and between infants in the non-tertiary care group and control group. These analyses required the formation of two matched groups (rather than three in the primary analysis) and therefore facilitated the formation of larger matched groups. With these analyses we tested the robustness of the primary findings in larger groups of infants, with different distributions of background data.

To evaluate the appropriateness of the selected caliper width, we matched infants using calipers of widths 0.05, 0.15, and 0.2 in addition to 0.1 and assessed the overall balance of background variables for each width. We also evaluated the impact of matching infants only on the propensity score, without additional matching on principal background variables.

### Patient and public involvement

Owing to the retrospective nature of the study utilising an existing research database, there was no direct involvement of patients or public. However, parents are informed of the inclusion of deidentified data on their infants into the NNRD and offered the opportunity to opt-out; to date no opt-out requests have been received. Parents are represented on the steering board of the Neonatal Data Analysis Unit, which oversees the NNRD. We will disseminate results through press releases, scientific meetings, social media, and directly to parents and families and professional and health policy organisations. Examples include Bliss, the UK charity for sick and preterm babies, the European Foundation for the Care of Newborn Infants, British Association of Perinatal Medicine, and the UK Maternal and Neonatal Health Policy Unit.

## Results

**Fig 1:**
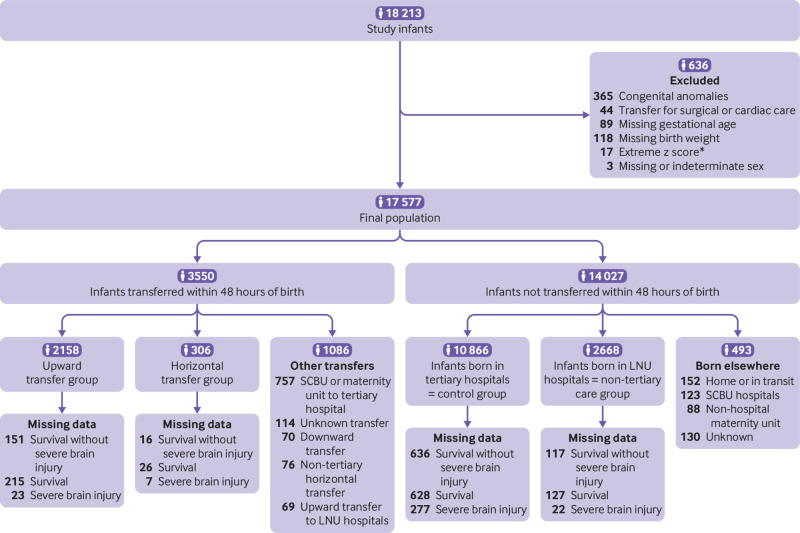
Flowchart of selection process of extremely preterm infants born less than 28 gestational weeks in England between 1 January 2008 and 31 December 2015. *74 infants born at less than 23 weeks without z score were matched based on sex, birth weight, and steroid use and were included in analyses. SCBU=special care baby unit; LNU=local neonatal unit

The population consisted of 18 213 extremely preterm infants. After exclusions and separately matching infants born before 23 weeks, 17 577 infants were retained (fig 1). A total of 3550 (20.2%) of the extremely preterm infants were transferred within 48 hours of birth. Early postnatal transfers increased from 18.4% in 2008 to 21.0% in 2015 (P=0.03). The proportion of infants born in hospitals with a local neonatal unit and not transferred within 48 hours declined from 21.4% in 2008 to 9.6% in 2015 (P<0.001). 

Of the 17 577 infants, 10 866 (61.8%) were in the control group, 2158 (12.3%) in the upward transfer group, 2668 (15.2%) in the non-tertiary care group, and 306 (1.7%) in the horizontal transfer group. The remaining 1579 infants were born in hospitals with either a special care baby unit or a maternity unit and transferred to a tertiary hospital (n=757), transferred downward to non-tertiary hospitals (n=70), born and remained in hospitals with a special care baby unit (n=123), had an unknown or unusual transfer pattern (transfer from a hospital with a special care baby unit or a local neonatal unit to a hospital with a local neonatal unit, n=259), or had an unknown or unusual place of birth (home, in transit, maternity units, out of country, n=370). Table 1 shows the background characteristics and standardised differences of the control, upward transfer, and non-tertiary care groups. The median gestational age was 25.6 weeks in the upward transfer group, 26.0 weeks in the control group, and 27.0 weeks in the non-tertiary care group.

The intervention groups were matched after propensity score assignment. The matching of infants in the upward transfer, non-tertiary care, and control groups yielded 727 triplets comprised of one infant from each group (2181 infants). The quality of the match is illustrated by the distribution of background variables and the standardised differences between the matched groups (table 2). The standardised differences after matching were smaller for all background variables compared with those in the unmatched groups (standardised differences ranging from 0.00 to 0.70 in the unmatched groups and from 0.00 to 0.068 in the matched groups). Supplementary table 1 displays summaries of all background variables that were used in the propensity analysis, and the standardised differences of the matched groups; standardisation is applied to this table so that the balances for the variables would be on compatible scales. Data on severe intraventricular haemorrhage were missing for at least one infant in 22 triplets, reducing the total number of comparable triplets to 705. Excluding triplets with missing data on survival yielded 571 triplets for analysis of death before discharge and 593 triplets for analysis of survival without severe brain injury.

**Table 2:** Background characteristics after propensity score matching of extremely preterm infants (<28 gestational weeks) born in England in 2008 to 2015, by hospital of birth and transfer status at 48 hours of age. Values are numbers (percentages) unless stated otherwise

Characteristics	Controls (n=727)	Upward transfer (n=727)	Standardised difference (matched)^*^	Non-tertiary care (n=727)	Standardised difference (matched)†	Standardised difference (matched)‡
Median (interquartile range) gestational weeks	26.0 (25.0-27.0)	26.0 (25.0-27.0)	0.000	26.0 (25.0-27.0)	0.000	0.000
Mean (SD) birth weight (g)	900 (56)	900 (69)	−0.012	888 (65)	0.015	0.027
Mean (SD) birth weight z score	0.099 (0.24)	0.103 (0.26)	−0.024	0.099 (0.25)	0.030	0.054
Boys	298 (41.0)	298 (41.0)	0.000	298 (41.0)	0.000	0.000
Multiple birth	158 (21.7)	162 (22.3)	−0.009	172 (23.7)	−0.033	−0.024
Missing	0	0		1		
Smoking in pregnancy	129 (20.1)	157 (24.1)	−0.068	146 (23.4)	−0.056	0.012
Missing	86 (11.8)	76 (10.5)		103 (14.2)		
Caesarean delivery	416 (57.2)	405 (55.7)	0.010	398 (54.7)	0.046	0.036
Missing	0	0		0		
Surfactant during resuscitation	701 (97.9)	695 (98.0)	−0.006	683 (95.9)	0.022	0.025
Missing	11 (1.5)	18 (2.5)		15 (2.1)		
Antenatal steroids	565 (80.5)	565 (78.9)	0.000	565 (81.2)	0.000	0.000
Missing	25 (3.4)	11 (1.5)		31 (4.3)		
Apgar score <3 at 1 min	144 (21.7)	144 (21.8)	−0.014	139 (20.9)	−0.016	−0.017
Missing	64 (8.8)	67 (9.2)		62 (8.5)		
Apgar score <3 at 5 min	34 (5.3)	34 (5.3)	−0.050	25 (3.8)	−0.049	0.005
Missing	84 (11.6)	82 (11.3)		74 (10.2)		

Upward transfer=infants born in hospitals with local neonatal units and transferred to tertiary hospitals within 48 hours of birth; non-tertiary care=infants born in hospitals with local neonatal units and not transferred within 48 hours of birth; control=infants born in tertiary hospitals and not transferred within 48 hours of birth.

* Controls versus upward transfer group.

† Controls versus non-tertiary care group.

‡ Upward transfer group versus non-tertiary care group.

Table 3 shows the estimated between group differences for the comparisons of upward transfer group with controls, non-tertiary care group with controls, and upward transfer group with non-tertiary care group. Compared with controls, infants in the upward transfer group had no significant difference in the odds of death before discharge (1.22, 95% confidence interval 0.92 to 1.61) but significantly higher odds of severe brain injury (2.32, 1.78 to 3.06) and significantly lower odds of survival without severe brain injury (0.60, 0.47 to 0.76). The NNT to prevent one case of severe brain injury was 8 (95% confidence interval 6 to 11) and to prevent one case of death or severe brain injury was 9 (6 to 17). Compared with controls, infants in the non-tertiary care group had significantly higher odds of death before discharge (1.34, 95% confidence interval 1.02 to 1.77) but no significant difference in the odds of severe brain injury (0.95, 0.70 to 1.30) or survival without severe brain injury (0.82, 0.64 to 1.05). The NNT to prevent one case of death was 20 (95% confidence interval 10 to 435). Compared with infants in the upward transfer group, infants in the non-tertiary care group had no significant difference in the odds of death before discharge (95% confidence interval 1.10, 0.84 to 1.44) but significantly lower odds of severe brain injury (0.41, 0.31 to 0.53) and significantly higher odds of survival without severe brain injury (1.37, 1.09 to 1.73). The NNT to prevent one case of severe brain injury was 8 (95% confidence interval 6 to 11) and to prevent one case of death or severe brain injury was 14 (8 to 58).

**Table 3:** Comparison of outcomes between propensity score matched extremely preterm infants (<28 gestational weeks) born in England in 2008 to 2015, by hospital of birth and transfer status within 48 hours

Outcomes	No (%; 95% CI)				Effect size % (95% CI)				Odds ratio (95% CI); P value		
	Upward transfer	Non-tertiary care	Controls		Upward transfer *v* controls	Non-tertiary care *v* controls	Non-tertiary care *v* upward transfer		Upward transfer *v* controls	Non-tertiary care *v* controls	Non-tertiary care *v* upward transfer
Death before discharge (n=571)	140 (24.5; (20.9 to 28.1)	150 (26.3; 22.6 to 30.0)	120 (21.0; 17.6 to 24.4)		3.50 (−1.47 to 8.47)	5.25 (0.23 to 10.27)	1.75 (−3.40 to 6.90)		1.22 (0.92 to 1.61); 0.16	1.34 (1.02 to 1.77); 0.04	1.10 (0.84 to 1.44); 0.50
Severe brain injury (n=705)	194 (27.5; 24.2 to 30.9)	95 (13.5; 10.9 to 16.1)	99 (14.0; 11.4 to 16.7)		13.48 (9.22 to 17.74)	−0.56 (−4.24 to 3.12)	−14.04 (−18.28 to −9.80)		2.32 (1.78 to 3.06); <0.001	0.95 (0.70 to 1.30); 0.76	0.41 (0.31 to 0.53); <0.001
Survival without severe brain injury (n=593)	338 (57.0; 42.9 to 61.1)	382 (64.4; 60.5 to 68.4)	408 (68.8; 65.0 to 72.6)		11.80 (6.21 to 17.39)	−4.38 (1.11 to −9.87)	7.42 (1.75 to 13.09)		0.60 (0.47 to 0.76); <0.001	0.82 (0.64 to 1.05); 0.11	1.37 (1.09 to 1.73); 0.009

Upward transfer=infants born in hospitals with local neonatal units and transferred to tertiary hospitals within 48 hours of birth; non-tertiary care=infants born in hospitals with local neonatal units and not transferred within 48 hours of birth; controls=infants born in tertiary hospitals and not transferred within 48 hours of birth.

To compare horizontal transfer and control groups, the propensity score matching was performed by matching each transferred infant to five controls, to exploit the relative abundance of infants in the control group. The groups were well matched on background variables (see supplementary table 2). This yielded 1525 matched control infants for the 305 horizontally transferred infants (table 4). Compared with controls, infants in the horizontal transfer group did not have a statistically significant difference in the odds of death before discharge (1.09, 95% confidence interval 0.80 to 1.42), severe brain injury (1.16, 0.83 to 1.54), or survival without severe brain injury (0.91, 0.71 to 1.15).

**Table 4:** Comparison of outcomes after pairwise matching extremely preterm infants transferred between tertiary hospitals within 48 hours of birth with non-transferred infants born in tertiary hospitals

Outcomes	No (%; 95% CI)		Effect size % (95% CI)	Odds ratio (95% CI)	P value
	Horizontal transfer (n=305)	Controls (n=1525)			
Death before discharge	64 (21.0; 15.4 to 26.8)	299 (19.6; 14.0 to 25.4)	1.41 (−4.39 to 7.20)	1.09 (0.80 to 1.42)	0.55
Severe brain injury	52 (17.0; 12.1 to 22.1)	230 (15.1; 10.1 to 20.1)	2.02 (−3.04 to 7.07)	1.16 (0.83 to 1.54)	0.36
Survival without severe brain injury	199 (65.2; 58.6 to 71.8)	1028 (67.4; 60.8 to 74.0)	−2.14 (−8.91 to 4.64)	0.91 (0.71 to 1.15)	0.43

Horizontal transfer=infants born in tertiary hospitals and transferred to another tertiary hospital within 48 hours of birth; controls=infants born in tertiary hospitals and not transferred within 48 hours of birth.

### Sensitivity analyses

To independently compare early postnatal transfer with controls, and ongoing non-tertiary neonatal care with controls, we conducted matched pairwise (rather than matched triplet) analyses. These sensitivity analyses involved separately matching upward transferred infants with controls and non-tertiary care infants with controls; this approach resulted in larger matched groups for pairwise comparison. When comparing upward transfer and control groups, the propensity score matching yielded 1825 pairs of infants. After matching, infants who underwent upward transfer had no significant difference in the odds of death before discharge (1.06, 0.92 to 1.23) but significantly higher odds of severe brain injury (1.38, 1.19 to 1.60) and significantly lower odds of survival without severe brain injury (0.84, 0.74 to 0.96) compared with controls (table 5). The NNT to prevent one case of severe brain injury was 18 (95% confidence interval 12 to 33) and to prevent one case of death or severe brain injury was 24 (14 to 105). When comparing non-tertiary care with control groups, propensity score matching yielded 2519 matched pairs of infants. Infants in the non-tertiary care group had higher odds of death before discharge (1.33, 95% confidence interval 1.19 to 1.49), no significant difference in the odds of severe brain injury (1.00, 0.88 to 1.14), and lower odds of survival without severe brain injury (0.81, 0.73 to 0.89) compared with controls (table 6). The NNT to prevent one case of death was 24 (95% confidence interval 15 to 57) and to prevent one case of death or severe brain injury was 24 (14 to 73).

**Table 5:** Sensitivity analysis: pairwise matching of extremely preterm infants born in non-tertiary hospitals and transferred to tertiary hospitals within 48 hours of birth (upward transfer group) compared with non-transferred infants born in tertiary hospitals (control group)

Outcomes	No (%; 95% CI)		Effect size % (95% CI)	Odds ratio (95% CI)	P value
	Upward transfer (n=1825)	Controls (n=1825)			
Death before discharge	523 (28.7; 26.6 to 30.8)	500 (27.4; 25.3 to 29.5)	1.30 (−1.68 to 4.28)	1.06 (0.92 to 1.23)	0.38
Severe brain injury	471 (25.8; 23.9 to 27.8)	368 (20.2; 18.4 to 22.0)	5.68 (3.03 to 8.31)	1.38 (1.19 to 1.60)	<0.001
Survival without severe brain injury	1025 (56.2; 53.9 to 58.4)	1101 (60.3; 58.1 to 62.6)	−4.16 (−7.36 to −0.96)	0.84 (0.74 to 0.96)	0.009

Upward transfer=infants born in hospitals with local neonatal units and transferred to tertiary hospitals within 48 hours of birth; controls=infants born in tertiary hospitals and not transferred within 48 hours of birth.

**Table 6:** Sensitivity analysis: pairwise matching of extremely preterm non-transferred infants born in non-tertiary hospitals with local neonatal units (non-tertiary care group) compared with non-transferred infants born in tertiary hospitals (control group)

Outcomes	No (%; 95% CI)		Effect size % (95% CI)	Odds ratio (95% CI)	P value
	Non-tertiary care (n=2519)	Controls (n=2519)			
Death before discharge	529 (21.0; 18.5 to 23.5)	421 (16.7; 14.2 to 19.2)	4.29 (1.77 to 6.81)	1.33 (1.19 to 1.49)	<0.001
Severe brain injury	327 (13.0; 10.9 to 15.2)	328 (13.0; 10.8 to 15.2)	−0.03 (−2.23 to 2.18)	1.00 (0.88 to 1.14)	0.98
Survival without severe brain injury	1753 (69.6; 66.8 to 72.5)	1864 (74.0; 71.1 to 76.8)	−4.32 (−7.26 to −1.37)	0.81 (0.73 to 0.89)	<0.001

Non-tertiary care=infants born in hospitals with local neonatal units and not transferred within 48 hours of birth; controls=infants born in tertiary hospitals and not transferred within 48 hours of birth.

The effect of using different caliper widths was evaluated (0.05, 0.1, 0.15, and 0.2) for propensity score matching. The rates of outcomes were robust to changes in caliper width (see supplementary table 3). Contrary to expectations, the balance between upward transfer and non-tertiary care groups was improved with increasing caliper width, but the balance between controls and the other groups declined with increasing caliper width. The rates of outcomes changed only marginally. The caliper width of 0.1 was retained. The balance of background variables was evaluated also using only propensity scores; this did not yield a superior match compared with matching on both propensity score and principal variables and did not result in materially different rates of outcomes across the different groups (see supplementary table 4).

## Discussion

By applying a robust matched analysis we found that extremely preterm infants born in a site without tertiary neonatal care had a higher risk of adverse outcomes. These results are from a high income, centrally funded, national healthcare system with high uptake of antenatal steroids and routine use of specialised neonatal transfer services. This association between non-tertiary birth and adverse outcomes is seen in both infants who underwent early postnatal transfer and infants who remained in a non-tertiary neonatal hospital.

### Strengths and limitations of this study

Our study has notable strengths. We prespecified our protocol and analysis plan and focused on the objective, clinically important outcomes, death and severe brain injury.^34^ We used individual data from a population level dataset of more than 17 000 extremely preterm infants, making this one of the largest studies to date. We applied a robust approach, using propensity score matching for multiple treatments and matching additionally on predefined principal variables to account for measured confounders. The formed groups were well balanced for distributions of covariates, and independently matched pairwise sensitivity analyses confirmed our findings. The predefined principal variables for matching were sex, gestational age, and use of antenatal steroids, all of which have a major impact on outcome.^41^ The final matched groups were similar for all measured background variables.

The main limitation of this study is that propensity score matching does not account for unmeasured confounders. This limitation could be dealt with by an instrumental variable approach, but we were unable to identify a suitable instrument in the available data. However, the features of propensity score matching enable reliable comparisons using observational data by eliminating the chance for errors resulting from false assumptions of the nature of background variables, and eliminating the temptation of selecting the most attractive statistical model.^36^ ^42^ We used all available pretreatment variables in the propensity score, as suggested previously.^43^

Through the application of careful matching we have limited the potential for measured confounders to influence the result. Nevertheless, important unmeasured factors need to be considered, including type and management of obstetric conditions, and stillbirths and delivery room deaths. In the analyses we did not match for obstetric variables such as placental disorders, presentation, and pre-eclampsia because they are not consistently recorded in the NNRD. The type and management of obstetric factors impact on neonatal outcomes and might have influenced our results. However, because complicating obstetric conditions are associated with worse neonatal outcomes^44^ and are concentrated in tertiary hospitals, their exclusion from the analysis is likely to lead to an underestimate of any benefit associated with birth in a tertiary hospital.^45^ A further limitation of this study is the exclusion of stillborn infants and those who died in the delivery room. This was unavoidable because the NNRD is primarily designed to hold data on neonatal admissions and has incomplete data on stillbirths and delivery room deaths. Because initiation of intensive care for the most extremely preterm infants is more common in tertiary hospitals,^46^ the most compromised and extremely preterm infants, with the highest rates of adverse outcomes, were more likely to have survived to admission in tertiary hospitals. Therefore any bias introduced by the exclusion of deaths in the delivery room is likely to be in the opposite direction to our findings and in favour of non-tertiary hospitals. We also recognise that we were unable to identify infants who underwent in utero transfer to tertiary hospitals before delivery because these data are not systematically captured in the NNRD. This would have permitted comparison between in utero and postnatally transferred infants.

### Interpretation of the findings

Our findings indicate that birth in a non-tertiary hospital and early postnatal transfer are associated with an increase in death and severe brain injury, even in the context of specialised neonatal transport services and advanced neonatal care. This suggests that approaches to reduce preterm mortality and morbidity^47^ should focus on ensuring, whenever possible, that extremely preterm infants are born in a setting with tertiary neonatal care.^48^ These data also indicate room for improvement in the current English networked model of neonatal care, as the median gestational age before matching in the upward transfer group was lower than in the control group. This indicates that many extremely preterm infants are not born in a tertiary hospital.

Extremely preterm infants contribute disproportionately to both neonatal deaths and perinatal brain injuries.^34^ Our study indicates that prioritising near universal delivery of extremely preterm infants in a setting with tertiary neonatal services, as found in many other high income countries,^17^ ^49^ ^50^ is likely to reduce neonatal death and brain injury.^35^ The rate of severe brain injury was not significantly higher in non-transferred infants who remained in non-tertiary neonatal hospitals compared with controls. This finding has two possible explanations: these infants might have died before severe brain injury was detected, as mortality was higher in this group, or there may be a mechanistic link between early postnatal transfer and brain injury related to physiological instability or vibration injury during transport.^51^

### Results in the context of other studies

Our results are in accord with previous work, including a study of 67 596 very low birth weight infants born in the USA between 1997 and 2004. This study concluded that postnatal transfer within 48 hours was associated with a higher rate of intraventricular haemorrhage (all grades).^24^ However, the study had several limitations. Infants were identified by birth weight rather than gestational age even though decisions about antenatal transfer tend to be guided by gestational age. As a result, infants small for gestational age—recognised to have a higher prevalence of neonatal morbidities, are likely to have been overrepresented in the transfer group, potentially biasing the results. Furthermore, potential perinatal confounders, such as use of antenatal steroids were not included in the analysis, whereas postnatal diagnoses (eg, necrotising enterocolitis) were included as confounders, even though they might have occurred after the intervention (transfer) and could potentially have been related to transfer. We avoided these limitations by forming matched groups based exclusively on factors that preceded early postnatal transfer and including gestational age and use of antenatal steroids, which have a strong influence on outcome.

Our study included the comparison of infants transferred between tertiary hospitals. This analysis was aimed at separating any possible effects relating to early postnatal transfer from delivery room care and initial stabilisation at a non-tertiary hospital. Three studies attempted similar comparisons,^52^ ^53^ ^54^ all single centre studies with small sample sizes, and two were undertaken more than 20 years ago. We did not find a statistically significant detrimental association between horizontal transfer and outcomes, but this result should be treated with caution. Although our study included the largest described population of horizontally transferred infants, the size of this group was small, comprising only 306 infants in an eight year period.

### Conclusions and policy implications

Extremely preterm birth in a non-tertiary neonatal setting is associated with a higher risk of death and lower survival without severe brain injury compared with infants born in a tertiary neonatal setting. These findings are in the context of specialised neonatal transfer services, high uptake of antenatal steroids, and the application of evidence based perinatal care. This has important policy implications for perinatal health services, indicating that care pathways that promote the birth of extremely preterm infants in hospitals with tertiary perinatal facilities should be considered a priority.

**What is already known on this topic**

Early postnatal transfer of extremely preterm infants between hospitals increased in England after the introduction of a networked model of neonatal care
In the context of modern care and specialised transfer teams, the association between early postnatal transfers and neonatal outcomes is not known

**What this study adds**

In extremely preterm infants, birth in a non-tertiary hospital is associated with an increased risk of death, and transfer in the first 48 hours is associated with an increased risk of severe brain injury, compared with infants born in hospitals with tertiary neonatal care who are not transferred in the first 48 hours
Perinatal services should promote pathways that facilitate delivery of extremely preterm infants in tertiary hospitals in preference to postnatal transfer

## Web extra

Extra material supplied by authors

**Supplementary information:** List of congenital malformations, covariates, details of matching, supporting tables, and sensitivity analyses

## Notes

Contributors: All authors contributed to the study planning. NL conducted the statistical analysis and all authors contributed to the interpretation of data. KH drafted the initial manuscript and NL, LL, NM, and CG reviewed the manuscript for intellectual content. All authors approved the final version of the manuscript for publication. NM, LL, and CG acted as guarantors and all authors accept full responsibility for the work and the conduct of the study. KH, CG and NL had full access to all the data in the study and take full responsibility for the integrity of the data and accuracy of the data analysis. The corresponding author attests that all listed authors meet authorship criteria and that no others meeting the criteria have been omitted.

Ethical approval: This study was approved by East Midlands-Leicester South research ethics committee (16/EM/0351) and approval for inclusion of the data in this study was obtained from all English neonatal units.

Data sharing: Data are available through the National Neonatal Research Database with relevant approvals (www.imperial.ac.uk/neonatal-data-analysis-unit/neonatal-data/utilising-the-nnrd/).

The guarantors (NM, LL, and CG) affirm that the manuscript is an honest, accurate, and transparent account of the study being reported; that no important aspects of the study have been omitted; and any discrepancies from the study as planned have been explained.
