# Supplementary material for: Association of early postnatal transfer and birth outside a tertiary hospital with mortality and severe brain injury in extremely preterm infants: observational cohort study with propensity score matching
Source: BMJ. 2019 Oct 16;367:l5678. doi: 10.1136/bmj.l5678 (PMC6812621; doi:10.1136/bmj.l5678)
Supplement: Supplementary file 1 — Supplementary information: List of congenital malformations, covariates, details of matching, supporting tables, and sensitivity analyses [file helk050424.ww1.pdf]

Supplement

List of contents:

Section 1. List of ICD-10 diagnostic codes used as exclusion criteria: severe congenital malformations requiring early surgical intervention

Section 2. Description of data items used for defining covariates and outcomes

Section 3. Description of the matching process

Section 4. Sensitivity analyses

Section 1. List of ICD-10 diagnostic codes used as exclusion criteria: severe congenital malformations requiring early surgical intervention

Q00	Anencephaly and similar malformations
Q01	Encephalocele and similar malformations
Q05	Spina bifida and similar malformations
Q20	Congenital malformations of cardiac chambers and connections
Q21.91	Single atrium
Q21.92	Single ventricle
Q21.2	Atrio-ventricular septal defect (AVSD)
Q21.3	Tetralogy of Fallot
Q22	Congenital malformations of pulmonary and tricuspid valves
Q23	Congenital malformations of aortic and mitral valves
Q25.1	Coarctation of aorta
Q25.2	Hypoplasia of aortic arch
Q25.3	Stenosis of aorta (AS)
Q25.4	Malformation of aorta
Q25.5	Atresia of pulmonary artery
Q25.6	Stenosis of pulmonary artery (PS)
Q25.8	Other congenital malformations of great arteries
Q25.8	Transposition of the great vessels (TGA)
Q26.2	Total anomalous pulmonary venous drainage (TAPVD)
Q39	Oesophageal atresia
Q41	Congenital absence, atresia and stenosis of small intestine
Q42	Congenital absence, atresia and stenosis of large intestine
Q60.1	Bilateral renal agenesis
Q60.6	Potter's syndrome
Q61.1	Polycystic kidney, infantile type
Q61.2	Polycystic kidney, adult type
Q64.1	Exstrophy of urinary bladder
Q64.2	Posterior urethral valves (PUV)
Q64.5	Congenital absence of bladder and urethra
Q77.1	Thanatophoric short stature
Q79.0	Congenital diaphragmatic hernia
Q79.1	Eventration of diaphragmatic hernia
Q79.2	Exomphalos
Q79.3	Gastroschisis
Q90	Down's syndrome
Q91	Edwards' syndrome and Patau's syndrome

Section 2. Description of data items used for defining covariates and outcomes

Covariates:

a) Z-scores were derived from UK growth standards using infant sex, gestational age at birth, and birth weight (Cole TJ et al. *Ann Hum Biol.* 2011)

For infants born below 23 gestational weeks there is no national standard; these infants were matched separately using sex, exposure to antenatal steroids and birth weight, as described in the Methods section in the main text.

b) Multiplicity was determined as singleton or any order of multiples using the variable "Number of Fetuses".

c) Maternal smoking was determined using the variable "Mother current smoker at booking".

d) Mode of delivery was determined using the variable "Mode of delivery", caesarean section denoting elective or emergency caesarean section, and vaginal delivery denoting spontaneous or instrumentally assisted vaginal delivery.

e) Exposure to antenatal steroids was determined using the variables "Steroids given during pregnancy to mature fetal lungs" and "Antenatal steroid course completion status". If either of these variables indicated that any antenatal steroids were administered during pregnancy, the infant was coded as "exposed to any antenatal steroids", and as "not exposed to antenatal steroids" only if both variables indicated so. If there

were no entries in either variable, the infant was coded as "unknown" regarding exposure to antenatal steroids. From these variables it is not possible to determine the exact timing of antenatal steroid administration before delivery, i.e. a mother recorded as given a complete course of antenatal steroids could have received the second dose at any time point before delivery.
f) Network where the delivery hospital was situated was determined using the variable "Site/organization code of actual place of delivery". The hospital codes and network codes are maintained by the NNRD staff, and not delivered to the researchers for data protection reasons.
g) The covariate "surfactant administered in the delivery room" was determined only by the variable "Surfactant given (during resuscitation)" and did not include any surfactant administered after admission to the neonatal intensive care unit.

Outcomes:
a) Death before discharge from neonatal care was determined using two variables; "Destination on discharge from neonatal critical care" and "Transferred for further care type". If the infant was discharged "Home", to "Ward (in the same organization)", or to "Foster/social care", the infant was coded as "survived to discharge from neonatal care". Infants who were recorded as transferred for surgical or cardiac care within 48 hours of age were excluded from the study. If the last known discharge destination was other than "Home", "Ward", "Foster/Social care" or "Died", survival to discharge was coded as "unknown".
b) Severe brain injury was defined as any recording of grade 3 or 4 intraventricular haemorrhage, porencephalic cyst, posthaemorrhagic hydrocephalus or cystic periventricular leucomalacia. These were identified using the episodic variables for cranial ultrasound findings, and additionally identified through searching for ICD-10 codes P91.2 (PVL) and P52.2 (grade 3 IVH), P52.3 (grade 4 IVH) recorded at any time before or upon discharge from neonatal critical care.
c) Survival without severe brain injury was determined by the absence of severe brain injury and infant surviving to discharge from neonatal care, as defined above.

Section 3. Description of the matching process

The subject of this study is a set of interventions in the care paths of babies born extremely preterm in their first two postnatal days. The interventions are:

1. *Upward transfer* from a non-tertiary to a tertiary unit within 48 hours from birth;
2. *Non-tertiary care*, birth in a hospital with non-tertiary unit and no transfer within 48 hours from birth;
3. *Horizontal transfer* from one tertiary unit to another within 48 hours from birth.

From a clinical point of view, these interventions are a priori second-rate to the recommended treatment, the *control*, which is birth in a tertiary unit and no transfer from it. The assignment to these three intervention groups is outside our control, and combines clinical and logistic considerations and is compounded by formal or informal arrangements and procedures of the units. The groups *upward transfer*, *non-tertiary care*, and *control* are compared in a single analysis.

For each baby we have an outcome, such as survival status. Babies with missing outcomes are excluded from the study. An extensive set of background variables is recorded. For variables with many missing entries a default value is imputed. For variables with appreciable rate of missing entries a separate category indicating nonresponse is defined. Variables for which a majority of entries are missing are discarded. All the variables are extracted from the NNRD. See Table 1 in the main manuscript for their summary.

Analysis
In the analysis, we seek matches for the babies with the *upward transfer* path among babies with paths *non-tertiary care* and *controls*, intending to form a set of triplets, with each path represented in a triplet by one baby, and no babies are included in more than one triplet. The triplets, reconstituted as three subsets of the intervention (path) groups, would then be analysed by a method that would appropriate in a linked randomised study. This analysis would compare the rates of positive outcomes for the *upward transfer* path vs. *controls, non-tertiary care* vs. *controls,* and *upward transfer* vs. *non-tertiary care*, using the same subset of babies with the upward transfer path. The rates would be compared by the t test.
Thus, the analysis comprises the following steps:

1. logistic regression analyses to obtain fitted propensities;
2. forming matched triplets based on the propensities;
3. analysis of the outcomes for the matched triplets.

The *horizontal transfer* group is much smaller than the other groups, thus horizontally transferred infants were separately matched to controls in a similar way, based on propensity scores and GA (weeks), sex, and exposure to antenatal steroids. To strengthen the analysis we matched each infant from the *horizontal transfer* group to 5 infants form the *control* group.

Propensity analysis

The propensity analysis entails two logistic regression analyses, one to obtain the propensities for the *upward transfer* path and the other for the *non-tertiary care* path (or *control* path). Thus, each baby has three propensities, one for each path; they add up to unity, so only two of them have to be estimated. We give details of the analysis for the *upward transfer* path.

The methods are drawn from two references:

McCaffrey, DF, Griffin, BA, Almirall, D, Slaughter, ME, Ramchand, R, and Burgette, LF. (2013). A tutorial on propensity score estimation for multiple treatments using generalized boosted models. Statistics in Medicine 32, 3388-3414. [Aspects of propensity analysis for three alternative treatments.]

Imbens, GW, and Rubin, DB. (2015). Causal Inference for Statistics, Social and Biomedical Sciences. Cambridge University Press, New York. [Aspects of propensity analysis and matching.]

The propensity analysis entails a logistic regression of the *upward transfer* path regarded as the positive outcome and the *non-tertiary-* and *control* paths as negative. The covariates are selected from the list of background variables and their interactions.

Main effects

The analysis starts with selection of a set of background variables included in the model a priori. These variables are: Intercept, Gestational age (weeks), Z score (for birth weight), Steroids (0/1), Surfactant (0/1), and Sex (M/F)

[This set of variables is different from the variables used to define the background groups with inflexible matching: 5 GA groups, Steroids and Sex.]

This logistic regression is fitted and is followed by fitting regressions with one other background variable added at a time. The variable with the highest absolute value of the t ratio is included, if this value exceeds 1.0. This cycle is repeated with the background variables as yet not included in the model; they are referred to as the candidate variables. The cycles are terminated when the t ratios of all the remaining candidate variables are smaller than 1.0 in absolute value, or when there are no candidate variables left. This analysis concluded with 22 covariates.

Interactions

This model of main effects is expanded by interactions. The covariates in the model are sorted according to the absolute values of their t ratios in the concluding model. This model is expanded by the interactions of the first covariate with one other covariate at a time. Two of these interactions with the highest absolute values of their t ratios are retained in the model, so long as these values exceed 2.71. If only one value exceeds 2.71, then only this one interaction is retained in the model. This procedure is then successively applied to all the other covariates, and their interactions, in the set order (more significant covariates have a precedence). This part of the analysis concludes with the addition of 36 interactions in the model.

Trimming

The set of all babies in the analysis is now reduced by discarding babies with extreme propensities (very close to zero or to unity). Two causes are pursued by this:

a) extreme propensities are a cause of poor fit of the propensity model and are a distraction in the matching process;

b) extreme propensities inflate the variation of the causal estimates.

We follow the proposals made in Imbens and Rubin, Chapter 13. For a), the dataset is reduced to the babies in the overlap of the propensities of the two groups. For b), a formula yields the upper bound for the smallest and a lower bound of the largest propensities. It suffices to implement b), because it discards all

the babies that would be discarded by a). This reduction, called trimming, reduces the dataset to 16,431 babies; their paths are
Upward transfer: 2303
Non-tertiary: 2731
Control: 11397
The propensity model is now fitted to this reduced dataset, using the same process as for the original set of babies. That is, the main effects are selected first, starting with the same set of a priori important variables, following by adding variables one-by-one, and then adding interactions. The concluding propensity model contains 22 main effects and 36 interactions, the same counts as for the models obtained in the first step. The set of main effects is unchanged, but there are a few differences among the interactions.
Trimming reduces the set of babies to 13,069; all babies with fitted propensities smaller than 0.0608 or greater than 0.9392 are excluded. None of the propensities exceed 0.9392, so only the bound for small propensities is relevant. The trimmed dataset comprises 1864, 2174 and 9031 babies with the respective paths *upward transfer*, *non-tertiary care*, and *controls*.
Similar (propensity) analysis is conducted for the *non-tertiary care* path as the focal intervention. It results in a reduction of the set of babies to 1152, 2558 and 5720 for the respective paths *upward transfer, non-tertiary care,* and *controls*. Here the trimming is much more severe, not because of the limits are extreme, 0.801 and 0.9199, but because many more propensities are extreme. In particular, many babies with *control* path are excluded.

Matching

To form matched triplets we reduce the set of babies to those who were retained by both propensity analyses. This results in a set of 6816 babies; they comprise 1051, 1676 and 4089 babies with respective paths *upward transfer*, *non-tertiary care*, and *controls*. Every baby has two propensities, one related to the *upward transfer* path and the other to the *non-tertiary care* path. We can also define a propensity for the *control* path; it is equal to the complement of the former two propensities: $P_{control} = 1 - P_{non\text{-}tertiary} - P_{upward}$, with a provision when this results in a negative value (only one baby is affected). The propensity scores are defined as the logits (that is, log-odds) of the propensities.
We apply caliper matching based on propensities. We set the caliper width to 0.10 but repeat the analysis for 0.05, 0.15 and 0.20 to confirm that the results do not differ substantially as the caliper size is changed. The role of the caliper is made obvious from the following description.
For each baby in the focal group (*upward transfer*), referred to as the focal baby, we define two sets of candidates for matching:
candidates with *non-tertiary care* path
and
candidates with *control* path
A candidate has to satisfy the following conditions
1. its propensity score (logit of the propensity) differs from the propensity score of the focal baby by less than the caliper width.
2. belong to the same background group defined by GA (5 groups), Steroids and Sex.
(The GA groups are defined by week, 23 -- 27, with babies born before 23 weeks included in the GA group 23.) Note that GA is also included among the covariates in the propensity model, where it is a continuous variable, with each day counted as 1/7 = 0.143 weeks.)
If these two conditions are satisfied by more than 50 babies, then the set of candidates is reduced to the 50 babies with the smallest difference of propensity scores. Each set is sorted in the ascending order of the difference of propensity scores (distance).
A focal baby has two sets of candidates, one set with *non-tertiary care* path and the other with *control* path. The babies in the focal group are sorted in the increasing order of abundance of their candidates for matching. That is, at the top of the list are babies who have only one candidate in each set, followed by one candidate in one set and two, three, etc. candidates in the other set. In other words, the sorting (order) is first by the minimum of the two numbers of candidates and then by the size of the larger set of candidates. Note that many focal babies have 50 candidates in both sets/paths. In contrast, there are a fair number of focal babies who have no candidates with at least one of the paths. Further losses may occur because focal babies may share (or even have identical sets of) candidates.
For the caliper width of 0.10, the set of focal babies is reduced from 1051 to 975; 76 babies have no candidates in one of their two sets; 52 focal babies have no candidates with *non-tertiary care* path and 30

focal babies have no candidates with *control* path; six focal babies are in both these lists (52 + 30 - 6 = 76).
Triplets are now formed by the following process, applied separately for candidates with *non-tertiary care* path and *control* path. The first candidate is picked for every focal baby, but picks that are repeated (babies that have been selected earlier) are discarded because a candidate baby can be used only once. Next, the second candidates are picked for all focal babies who have at least two candidates and for whom the first candidate was discarded. A pick that has been used earlier is discarded. Then the third and successive candidates are picked until there are no candidates left for focal babies that as yet have no matches. At the conclusion of this process we obtain a list of matched triplets, with some positions in these triplets not occupied.
We obtained 727 complete triplets; 228 triplets have the position for *non-tertiary care* not occupied and 14 have the position for *control* not occupied. In six instances both positions are unoccupied, i.e. we failed to find a match with *non-tertiary care* and *control* paths.

Assessment of balance
The quality of the match is assessed by the differences of the rates for binary background variables and differences of the means for the continuous (ordinal) variables. These differences are scaled -- divided by the pooled standard deviation of the variable (pooled across the path/intervention groups) -- so that they would be on compatible scales. Note that these standardised differences differ slightly from the ones presented in Table 2 in the main manuscript; this is because Table 2 in the main manuscript is a direct comparison of the rates and differences among clinically important background variables after matching (compared to Table 1, which shows the unbalanced distribution of background variables before matching), and allows the reader to easily compare the attained match. Supplementary table 1 contains an exhaustive list of all variables used in the propensity analysis, pooled across groups to allow for comparison between individual variables on a compatible scale. In this sense, Supplementary table 1 is more sensitive to small differences in balance; see McCaffrey et al. for reference. There are 32 background variables, so the assessment has the form of a 32 x 3 table; one column for comparing upward transfer and control paths, one for comparing *non-tertiary care* and *control* paths, and one for comparing *upward transfer* and n*on-tertiary care* paths. For the caliper width of 0.10, we obtain the following table of balances:

Supplementary table 1. Background characteristics after propensity score matching of extremely preterm infants (<28 gestational weeks) born in England in 2008 to 2015 grouped by level of neonatal unit of birth and transfer status at 48 hours of age.

	Non-tertiary care vs. control	Upward transfer vs. control	Upward transfer vs. non-tertiary care
Admission Date	0.112	0.072	-0.041
Admission date^2	0.101	0.070	-0.031
Z-score	0.054	0.024	-0.030
Z-score^2	-0.052	-0.016	0.033
GA	0.034	0.015	-0.019
GA^2	0.034	0.015	-0.019
Sex	0.000	0.000	0.000
Multiple birth	-0.023	0.009	0.032
Smoking in pregnancy	0.026	0.069	0.042
Smoking in pregnancy missing	0.080	0.031	-0.049
Antenatal steroids	0.000	0.000	0.000
Vaginal delivery	0.031	-0.022	-0.053
Delivery mode missing	-0.027	-0.023	0.004
APGAR 1min	0.002	-0.015	-0.015
APGAR 5min	-0.036	-0.042	-0.005
APGAR 1min missing	-0.010	0.007	0.017
APGAR 5min missing	0.006	0.031	0.025
Surfactant	0.052	-0.030	-0.082

ODN1	-0.003	0.160	0.163
ODN2	0.067	0.037	-0.030
ODN3	0.024	0.055	0.031
ODN4	0.000	-0.069	-0.069
ODN5	0.076	0.020	-0.055
ODN6	-0.115	-0.035	0.083
ODN7	-0.092	-0.089	0.003
ODN8	---	-0.189	-0.189
ODN9	-0.029	-0.029	0.000
ODN10	0.005	-0.005	-0.011
ODN11	-0.046	-0.060	-0.014
ODN12	-0.014	-0.005	0.009
ODN13	0.039	0.044	0.005
ODN14	0.011	0.035	0.024

ODNs are indicators of networks (Operational Delivery Networks). The entry --- for ODN8 and *non-tertiary care* path indicates that among the matches with *non-tertiary care* path there is only one baby from this network. [For a sample with a single baby the standard deviation cannot be evaluated.] For Sex and Steroids, the balance is perfect because of exact matching. For GA (weeks) the balance is not perfect because the variable used in estimating the propensity differs from the variable used for exact matching (5 categories). According to McCaffrey et al (2013; Statistics in Medicine), a balance statistic in the range (-0.20, 0.20) raises no concern. In our case, all balances fall into this range, although one by a narrow margin (-0.189 for ODN8 and *upward transfer* vs. *control* path).

We can summarise the statistics in this table by their standard deviations, which are similar to the averages of their absolute values. These summaries are 0.0533 and 0.0593 for the respective paths *non-tertiary care* and *control*. The summary for the *control* path is affected by the balance for ODN8 although the balance is relatively large also for ODN1 (0.160).
This evaluation has one flaw, namely that further triplets are discarded because the outcomes are missing for some babies in the matched triplets. A triplet with a missing value is discarded from the analysis. [At this stage, the analysis has not involved the outcomes at all.]

Because the relatively small size of the *horizontal transfer* group did not allow for inclusion into the three-way matching, we matched these infants pairwise to controls. The balance of background variables both before and after matching is shown in Supplementary table 2 below.

Supplementary table 2. Background characteristics before and after pairwise matching extremely preterm infants transferred between tertiary units within 48 hours of postnatal age (horizontal transfer group) to non-transferred infants born in tertiary units (control group).

	Control (N=10,866)	Horizontal transfer (N=306)	Standardised difference (unmatched)	Standardised difference (matched)
Gestational weeks, median (IQR)	26.0 (24.9, 27.0)	26.3 (25.1, 27.1)	0.16	0.00
Mean birth weight, grams, (SD)	807 (188)	858 (189)	0.20	0.02
Birth weight z-score, mean (SD)	-0.20 (0.89)	-0.04 (0.80)	0.19	0.01
Male sex (%)	5,799 (53.4)	174 (56.9)	-0.07	0.00
Multiple birth (%)	2,995 (27.6)	115 (37.7)	-0.19	0.00

Missing values (%)	2 (0)	1 (0.3)		
Smoking in pregnancy (%)	1,733 (19.5)	43 (19.1)	-0.04	0.00
Missing values (%)	1,998 (18.4)	81 (26.5)		
Caesarean delivery (%)	4,028 (40.1)	125 (45.0)	-0.10	0.00
Missing values (%)	819 (7.5)	28 (9.2)		
Surfactant during resuscitation (%)	9,780 (94.0)	283 (97.6)	0.06	0.00
Missing values (%)	466 (4.3)	16 (5.2)		
Antenatal steroids (%)	9,897 (92.4)	276 (90.5)	-0.02	0.00
Missing values (%)	153 (1.4)	1 (0.3)		
Apgar 1 min <3 (%)	1,847 (19.5)	29 (11.6)	-0.04	0.00
Missing values (%)	1,392 (12.8)	57 (18.6)		
Apgar 5 min <3 (%)	385 (4.1)	7 (2.9)	-0.04	0.00
Missing values (%)	1,426 (13.1)	65 (21.2)		

IQR, Interquartile range; SD Standard deviation

Section 4. Sensitivity analyses

Although the caliper width of 0.10 is preferred by many, the arguments in its support are not profound; it is generally regarded as a pragmatic choice. We evaluate the estimates of the rates and means of the outcomes using matching with other caliper widths: 0.05, 0.15 and 0.20 in Supplementary table 3 below.

Supplementary table 3. Sensitivity analysis of estimates for rates of outcomes of extremely preterm infants using different caliper widths

	Controls % (SE)	Upward transfer % (SE)	Non-tertiary care % (SE)
Caliper width 0.05			
Severe brain injury (N=642)	14.02 (1.37)	26.48 (1.74)	13.24 (1.34)
Death before discharge (N=520)	17.69 (1.68)	22.12 (1.82)	25.58 (1.92)
Caliper width 0.1			
Severe brain injury (N=705)	14.04 (1.31)	27.52 (1.68)	13.48 (1.29)
Death before discharge (N=571)	21.02 (1.71)	24.52 (1.80)	26.27 (1.84)
Caliper width 0.15			
Severe brain injury (N=718)	14.62 (1.32)	28.27 (1.68)	13.65 (1.28)
Death before discharge (N=591)	20.98 (1.68)	25.38 (1.79)	25.72 (1.80)
Caliper width 0.2			
Severe brain injury (N=746)	15.28 (1.32)	26.01 (1.61)	13.81 (1.26)
Death before discharge (N=613)	20.88 (1.64)	24.14 (1.73)	25.94 (1.77)

SE, Standard error

These results confirm that the choice of the caliper width is not important; the same conclusions are arrived at for all four choices. With wider calipers we have larger sample sizes, but this is reflected in only marginal (and inconsequential) reduction of the standard errors. The number of triplets formed with the

respective caliper widths 0.05, 0.10, 0.15 and 0.20 are 661, 727, 744 and 772. Narrower calipers are preferred by many because of the promise of a better match. This is not born out in our study, since the overall balance of background variables was similarly excellent using caliper widths 0.05 and 0.10 (mean standardised difference 0.02 in both), but the balance of some variables was poorer with caliper width 0.05. Caliper widths of 0.15 and 0.20 yielded poorer balance (mean standardised difference 0.03 in both).

We repeated these analyses with matching on propensity only (ascribing no special status to GA, Steroids and Sex). A cosmetic advantage of these analyses is that their sample sizes in the final stage are greater. The results differ from their counterparts discussed above only slightly. For example, in Supplementary table 4 we show the results for severe brain injury and caliper width of 0.10, when matching on propensity score only. Of note is that the balance was equally excellent in both ways of matching (mean standardised difference 0.021 and 0.020), but the balance for some background variables were poorer using only propensity score.

Supplementary table 4. Incidence of severe brain injury in extremely preterm infants after matching on propensity score only.

N=816	Severe brain injury % (SE)
Upward transfer	27.45 (1.56)
Non-tertiary care	12.62 (1.16)
Controls	15.81 (1.28)

SE, standard error

For the contrast upward transfer vs. controls, we have the estimate 11.64 with standard error 2.02; the corresponding p value is smaller than 0.0001, and the balance on the background variables is not improved with this way of matching.
